# A novel method to evaluate the transverse pedicle angles of the lower lumbar vertebrae using digital radiography

**DOI:** 10.1371/journal.pone.0295196

**Published:** 2024-06-13

**Authors:** Shixun Wu, Shizhang Liu, Ming Ling, Minggang Huang, Zhe Liu, Xianglong Duan

**Affiliations:** 1 Department of Orthopedics Surgery, Shaanxi Provincial People’s Hospital, Xi’an, Shaanxi, China; 2 Key Laboratory of Bone Joint Disease Basic and Clinical Translation of Shaanxi Province, Xi’an, Shaanxi, China; 3 Department of Computed Tomography, Shaanxi Provincial People’s Hospital, Xi’an, Shaanxi, China; 4 Second Department of General Surgery, Shaanxi Provincial People’s Hospital, Xi’an, Shaanxi, China; 5 Institute of Medical Research, Northwestern Polytechnical University, Xi’an Shaanxi, China; 6 Second Department of General Surgery, Third Affiliated Hospital of Xi’an Jiaotong University, Xi’an, Shaanxi, China; Columbia University Vagelos College of Physicians and Surgeons, UNITED STATES

## Abstract

To investigate a novel approach for establishing the transverse pedicle angle (TPA) of the lower lumbar spine using preoperative digital radiography (DR). Computed Tomography (CT) datasets of the lower lumbar were reconstructed using MIMICS 17.0 software and then imported into 3-matic software for surgical simulation and anatomical parameter measurement. A mathematical algorithm of TPA based on the Pythagorean theorem was established, and all obtained data were analyzed by SPSS software. The CT dataset from 66 samples was reconstructed as a digital model of the lower lumbar vertebrae (L3-L5), and the AP length/estimated lateral length for L3 between the right and left sides was statistically significant (*P* = 0.015, *P* = 0.005). The AP length of the right for L4 was smaller than that of the left after a paired *t* test was executed (*P* = 0.006). Both the width of the pedicle and the length of the pedicle (P2C1) were consistent with TPA (L3<L4<L5). There were no significant differences in TAN-TPA and DR-TPA compared with real TPA. The ICCs for the real TPA and DR-TPA within L3 showed good reliability, and the ICCs for the real TPA and DR-TPA within both L4 and L5 showed moderate reliability. Our novel approach can be considered a reliable way to determine the transverse pedicle angle from routine DR, and the width and length of the pedicle within lumbar DR should be considered to determine the length and trajectory of the screw during preoperative planning.

## Introduction

Pedicle screw fixation technology is the standard method for maintaining the stability of the spine during the treatment of degenerative lumbar instability, spine tuberculosis [1], fracture, and septic spondylitis. Pedicle screws can supply stability for the three columns of vertebrae through the cortical bone of the pedicle [2].

Freehand pedicle screw fixation during the operation is a universal and acceptable approach; [3–5] however, a postoperative computed tomography (CT) study demonstrated that 3.9% of screw breach for freehand pedicle screw technology was identified [6]. Another similar publication demonstrated 13.5% of misplace screws [5].

Even though herringbone is based on relatively constant anatomical landmarks, it is mostly used to ensure the entry angle of the pedicle screw. Indeed, the TPA referring to herringbone is obviously less than the true TPA [7], which leads to the entry point being close to the facet joint and posterior midline, damaging the stability of the spine, and even adding to the possibility of inner wall rupture and nerve damage.

The new approaches, such as computer navigation and robotic assistance, improve the precision of pedicle screw implantation [8]. Conversely, some studies have reported that the accuracy of pedicle screw insertion between freehand technology combined fluoroscopy and intraoperative CT navigation is similar [9–11]. Similarly, navigation and robotic assistance increase operation time and cost [12, 13], and CT navigation and robotic assistance at present are controversial and difficult to popularly apply.

The deformation of the pedicle of the lumbar vertebra varies greatly. It is very important for preoperative planning to reduce the risk of neurological deficits and lower extremity weaknesses. Digital radiography (DR) is a routine radiological method before internal fixation for lumbar surgery. The method of simulating surgery based on three-dimensional reconstruction can be used to obtain more accurate anatomical information [14].

To the best of our current knowledge, the size of TPA predicted from the standard DR has not been published. In this study, a digital model of the vertebral body was established using CT data from human subjects, and the simulated operation of pedicle screw insertion was compared with simulated DR in an attempt to find a convenient and accurate method for evaluating the TPA using DR technology.

## Methods and materials

### Specimen acquisition and computer-assisted software

A SOMATOM Definition Flash dual-source CT machine (Siemens Healthineers, Forchheim, Germany) was selected to scan the lumbar vertebral bodies of the subjects, including the L3-L4-L5 body. The parameters were set as follows: 120 kV, 205.50 mAs, layer thickness: 1 mm, and all DICOM images (521 px×512 px) in 336 layers for each subject. All methods were performed in accordance with the relevant guidelines and regulations [15]. CT data were imported into Materialise’s Interactive Medical Image Control System (MIMICS) 17.0 software (Materialise, Leuven, Belgium), and the region of interest (ROI) was extracted using both the "Thresholding" and "region growing" modules. All 3D models of the lumbar spine were automatically produced by the "calculate 3D from mask" functional block and then imported into 3-matic software for surgical simulation and anatomical parameter measurement. The pedicle screw was designed using SolidWorks 2012 X 64 edition (Dassault Systems SolidWorks Corp., Waltham MA). The computer workstation included a Lenovo thinkpad, Windows 7–64 bit operating system, Intel (R) Core(TM) i7-4600 processor, 8 GB of running memory, and 256 SSD hard disk. All the subjects signed the participant consent form. This study was approved by the Ethics Committee of Shaanxi Provincial People’s Hospital.

The lumbar CT data from subjects were retrospectively collected in the outpatient and inpatient departments of Shaanxi Provincial People’s Hospital from May 2022 to May 2023. The inclusion criteria were as follows: patients without/with a history of trauma but without fracture or dislocation from the lower lumbar spine. The exclusion criteria were as follows: (i) the vertebral body of the patient was fractured, (ii) the patient had congenital or acquired skeletal deformity, and (iii) the patient had destruction of the vertebral bone caused by a tumor or infection (spinal tuberculosis).

### Calibration of the coordinate system

**Fig 1:**
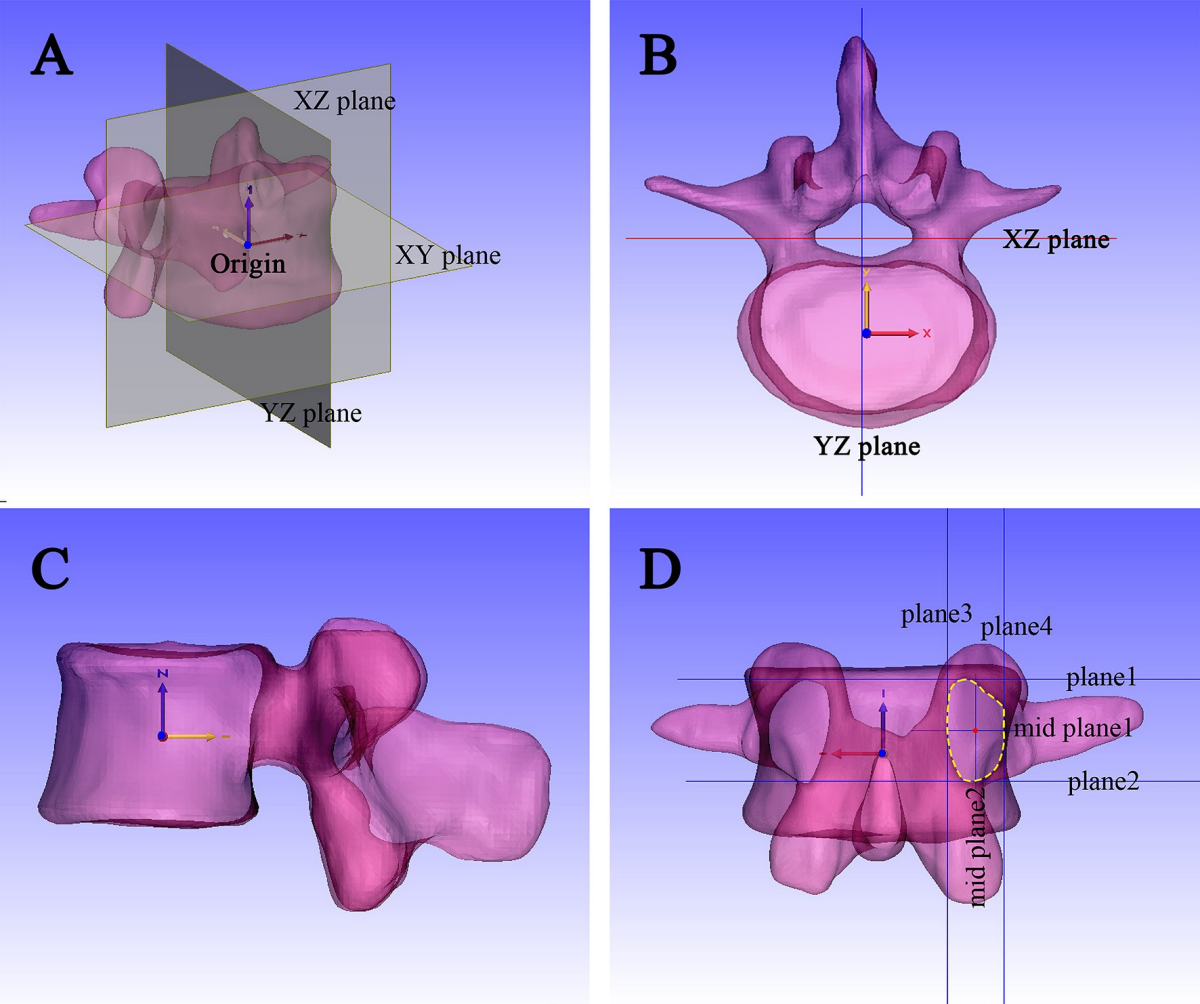
Establishment of standard coordinate system for lumbar model. (A) Establishment of the XY plane, YZ plane, and XZ plane; (B) Top view of the standard digital model; (C) Right view of the standard digital model; (D) Center point of the pedicle established by planes.

The object coordinate system (OCS) of the vertebrae bodies were formatted to the World coordinate system (WCS) to keep each vertebral body in the same three-dimensional position in the software. Origin point (0,0,0)was defined as the centerpoint of each vertebra (except attachment) in World coordinate system(WCS), XY plane (axial plane)、YZ plane (sagittal plane)、ZX plane (coronal plane) was generated separately (Fig 1A).

### Simulating DR of the standard vertebral body

The *Mark* function of 3-matic was used to separate the vertebral body and lumbar pedicle to calculate the center of gravity of the separated vertebral body. Then, both the center point and vertebral body were aligned with the origin point. On the top view, the upper surface of the vertebral body was aligned to the XY plane, and then translation and rotation functions were executed repeatedly to normalize the vertebral body, all according to the guidelines of standardized lumbar DR [16] (Fig 1B and 1C).

### Standardized lumbar DR

Anteroposterior view (AP): pedicles and transverse processes on both sides are symmetrical, and all the edges of the vertebral body overlap well with no double-layered wall. Lateral view: all the edges of the vertebral body overlap well with no double-layered wall (Fig 1B and 1C).

### Center point of the pedicle

**Fig 2:**
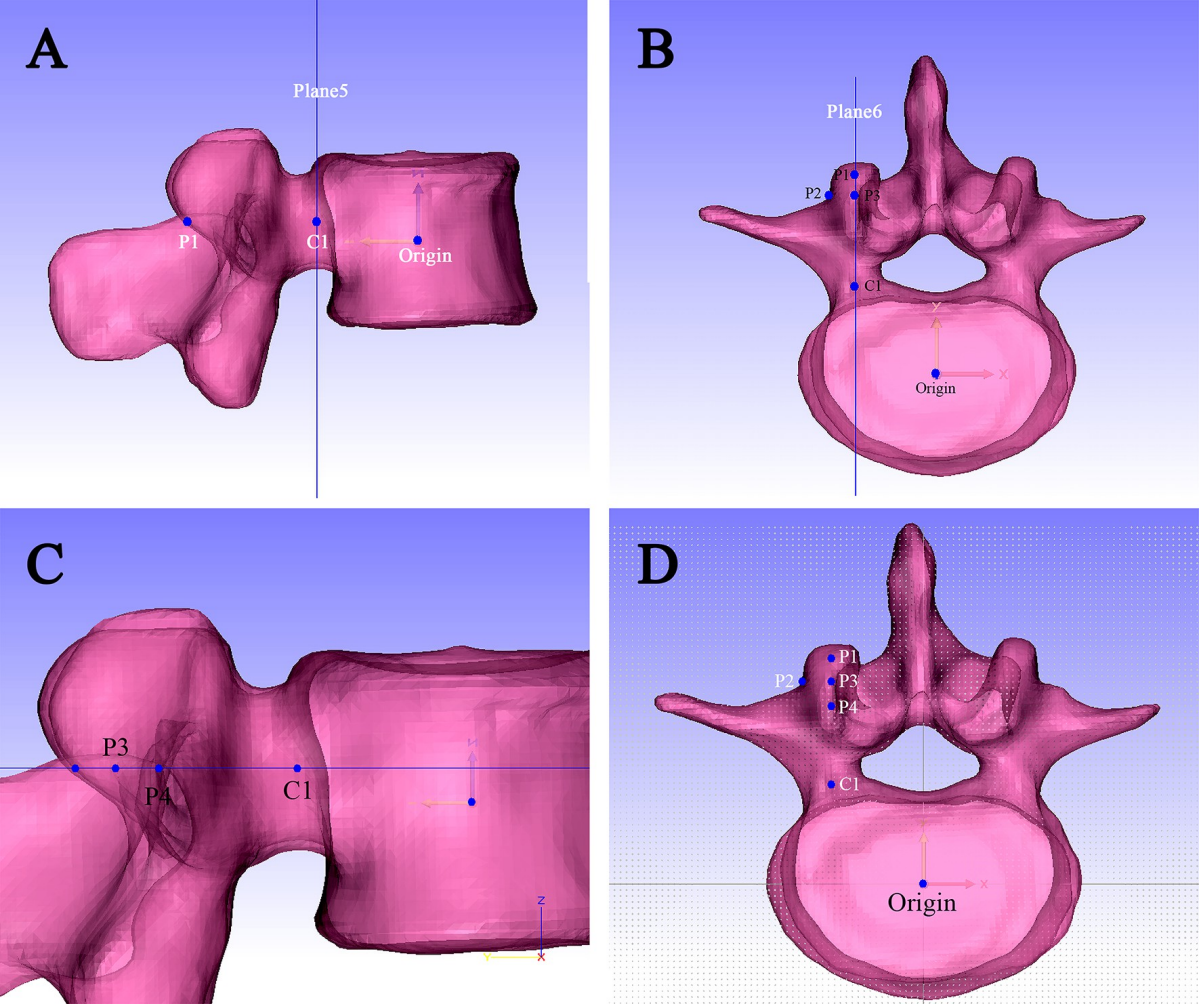
Establishment of five key points for this approach. (A) Determination of points P1 and C1; (B) Determination of points P2 and P3; (C) P4 is located in the color heaviest area of the transverse process; (D) The five points were projected onto a sketch.

On the back view, parallel to the XY plane, plane1 was positioned through the bottom of superior wall of pedicle; In the same approach, plane2 was positioned through the top of inferior wall of pedicle; Mid plane1 was positioned based on plane1 and plane2; In the same approach, parallel to the YZ plane, plane3 and plane4 were generated based on the inner and outer walls of the pedicle; and mid plane2 between plane3 and plane4 was generated. Line 1 (L1) was positioned by midplane1 and midplane2 (Fig 1D). The projection point onto the vertebra was created as point 1(P1), then P1 was aligned with articular process border shadow; A new plane5 parallel to the XZ plane was created through the top of inferior vertebral notches, center point (C1) of the pedicle is the point which P1 was projected to the plane5 (Fig 2A).

Line 2 (L2) was created by plane4 and midplane1, which was projected to the vertebra, and the projection point (P2) was the entry point of pedicle screw placement. Plane6 was created through the C1 point and parallel to the YZ plane, and Point 3 (P3) was set as the P2 projection onto plane6 (Fig 2B). Under the left view, the P3 point was duplicated and translated to the heaviest color area of the transverse process (P4) (Fig 2C).

### Measurement approach

A sketch was created on the XY plane in 3-matic, and then the five points (C1/P1/P2/P3/P4) were created correspondingly and projected onto this sketch (Fig 2D), and the distance between two points was measured.

### Algorithm of TPA

**Fig 3:**
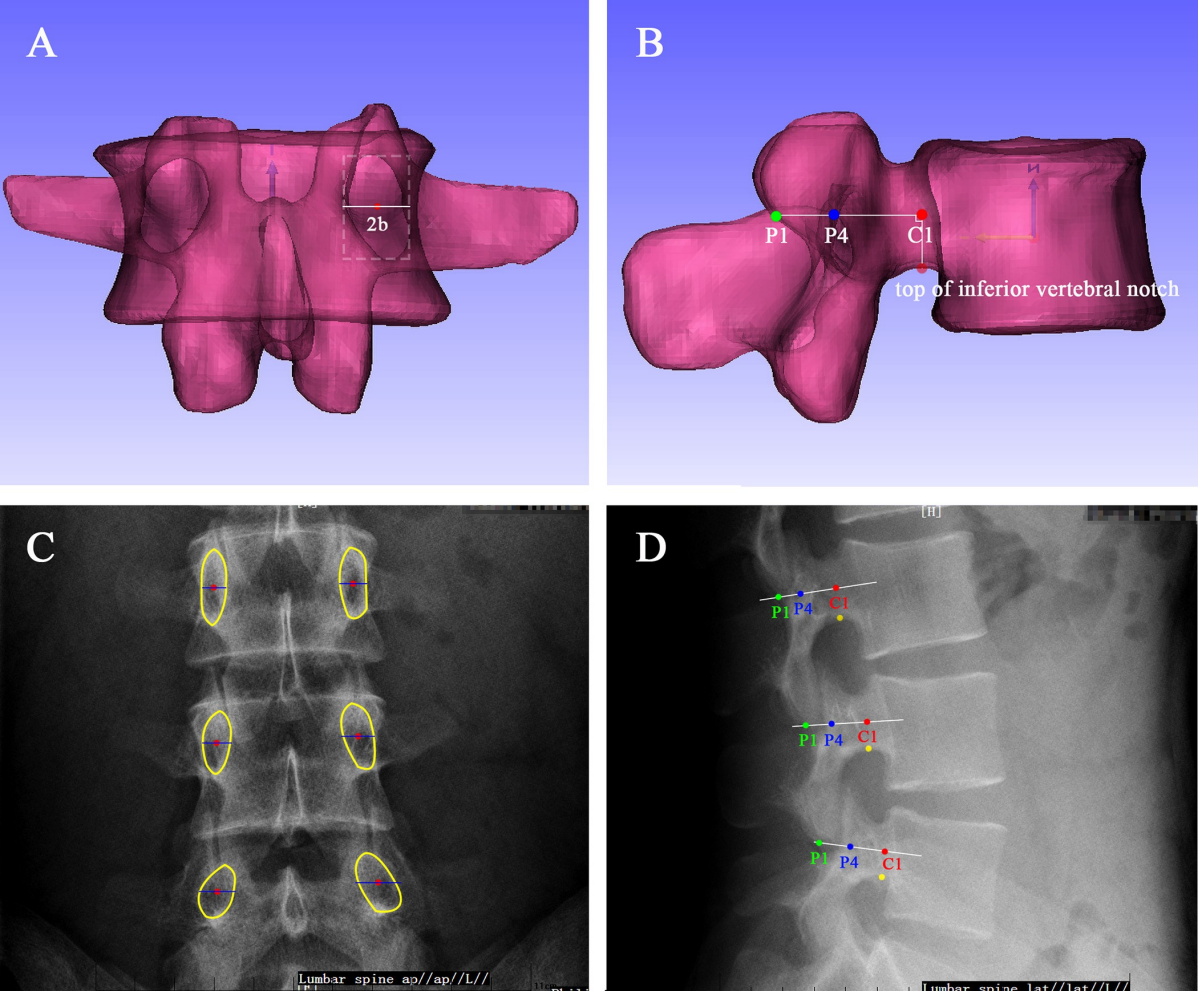
Measurement of TPA for the lumbar model and DR. (A) Pedicle width of the lumbar model under AP view; (B) Pedicle length of the lumbar model under lateral view; (C) Pedicle width of the DR under AP view; (D) Pedicle length of the DR under lateral view, yellow indicates the top of the inferior vertebral notch.

According to the definition of TPA published previously [17], the assessment method can be expressed as follows; Under the standard AP view, the width of the pedicle is marked as 2b, and the length of the pedicle under the lateral view is marked as L2, L2 = a2+a3. Then, the estimated formula of TPA is expressed as α≈ATAN(b/L2). Measurement diagrams in the lumbar model and DR are shown in Fig 3.

**Fig 4:**
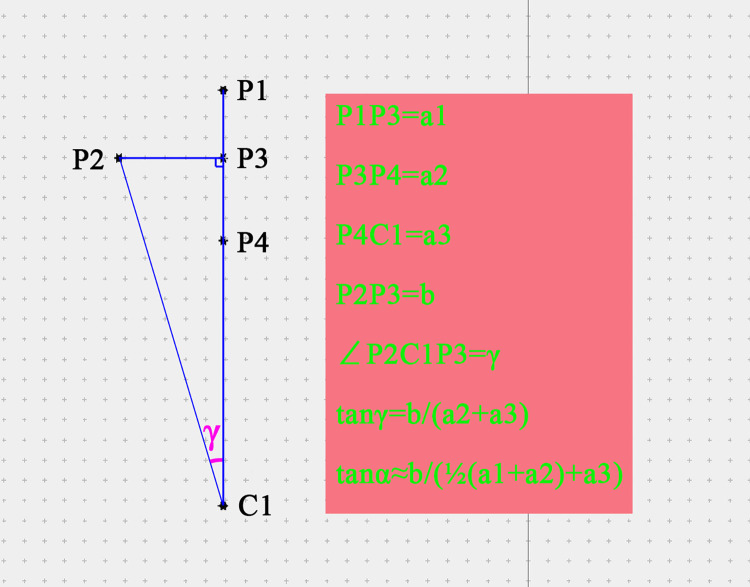
Schematic diagram of measurement length and TPA.

However, the length of the pedicle can be determined under the lateral view with the following formula: L2≈a3+ ½(a1+a2) (Fig 4)

TAN-TPA was expressed as α = DEGREES(ATAN(b/(a2+a3))

**DR-TPA** was expressed as **β = DEGREES(ATAN(b/L2))**

TPA (γ) was measured using 3-matic software.

### Statistical analysis

All measurements of the vertebral body in this study included the left and right sides, and each vertebral body was measured by the same orthopedic surgeon. All data were collected and entered into Microsoft Excel 2016, SPSS17.0 statistical software package (SPSS Statistics for Windows, Version 17.0. Chicago: SPSS Inc.) was employed to determine the statistical results. If the data were normally distributed and had equal variance, an independent sample t test was used to identify the difference between two groups. A paired *t* test was used to analyze the difference between the left and right pedicles, and the results are expressed as the mean ± standard error ($\bar{x}$±SEM). The difference among multiple samples was tested by the LSD multiple comparison method; if the three groups of data were not normal or the variance was unequal, the Kruskal‒Wallis H nonparametric test was used. The missing value was replaced by average value. The intraclass correlation coefficient (ICC) was used to test the consistency, all statistical tests were two-sided, and *P*<0.05 was considered statistically significant.

## Results

### Baseline information for CT data

According to the inclusion criteria, 66 samples were collected, including 29 males and 37 females, with an average age of 32.48±7.26 (16–57 years). All the results of measurement by 3-matic software are displayed in Tables 1–3.

**Table 1:** Baseline measurement data for the L3 vertebral body.

L3	N	Left				Right			
		mean	SD	min	max	mean	SD	min	max
Lateral length(mm)	54	17.11	2.27	12	22	16.81	2.44	11.23	22.55
AP length(mm)	54	4.8	0.72	3	8	4.69	0.68	2.50	6.41
a1(mm)	54	2.62	2.11	0.01	9.07	2.48	2.03	0.01	10.10
a2(mm)	54	4.51	1.76	1.36	8.17	4.26	2.03	0.80	8.29
a3(mm)	54	12.67	1.44	8.88	15.95	12.59	1.33	9.08	15.37
Estimated Lateral length(mm)	54	16.23	1.78	11.46	20.02	15.76	1.82	11.68	19.95
∠α (TAN-TPA)	54	15.99	3.24	9.39	25.75	15.97	3.51	7.15	28.19
∠γ (TPA)	54	15.99	3.24	9.38	25.76	16.43	4.81	7.15	42.70
∠β (DR-TPA)	54	16.61	2.63	11.16	24.67	16.50	2.54	8.67	22.93

**Table 2:** Baseline measurement data for the L4 vertebral body.

L4	N	Left				Right			
		mean	SD	min	max	mean	SD	min	max
Lateral length(mm)	66	17.62	3.77	10.05	25.10	17.72	3.66	11.56	27.42
AP length(mm)	66	5.58	0.84	3.82	8.69	5.43	0.77	3.84	7.14
a1(mm)	66	3.80	3.42	0.01	14.68	3.46	3.57	0.01	12.60
a2(mm)	66	6.08	3.38	0.52	14.68	6.43	3.58	0.75	14.84
a3(mm)	66	11.48	1.78	7.97	16.95	11.26	1.53	7.79	15.15
Estimated Lateral length(mm)	66	16.42	2.13	10.93	21.91	16.21	1.84	11.77	19.15
∠α (TAN-TPA)	66	18.35	5.23	10.85	32.11	17.70	4.56	10.60	29.57
∠γ (TPA)	66	18.35	5.22	10.84	32.12	17.70	4.55	10.60	29.58
∠β (DR-TPA)	66	18.93	2.95	12.44	25.96	18.66	2.98	13.34	27.54

**Table 3:** Baseline measurement data for the L5 vertebral body.

L5	N	Left				Right			
		mean	SD	min	max	mean	SD	min	max
Lateral length(mm)	66	18.17	3.32	12.02	24.58	18.05	4.27	8.61	26.53
AP length(mm)	66	6.64	0.93	4.66	8.84	6.56	1.03	4.57	9.49
a1(mm)	66	3.37	3.24	0.01	13.48	3.80	3.67	0.01	12.47
a2(mm)	66	5.65	3.22	0.51	12.32	5.83	3.35	0.21	12.50
a3(mm)	66	12.53	3.01	5.32	20.93	12.29	3.12	3.96	18.64
Estimated Lateral length(mm)	66	17.02	2.68	10.14	22.90	17.01	2.86	9.61	23.03
∠α (TAN-TPA)	66	20.56	4.22	13.17	29.50	20.92	5.82	13.09	35.32
∠γ (TPA)	66	20.56	4.21	13.17	29.47	20.89	5.81	13.10	35.31
∠β (DR-TPA)	66	21.52	2.94	16.26	28.43	21.38	3.55	14.20	32.42

### Testing of the standardized method for simulated digital radiology

A paired *t* test was employed, and the result for L3 showed that the AP length /estimated lateral length between the right and left sides was statistically significant (*P* = 0.015, *P* = 0.005). All other variables were not significant. For L4, the AP length of the right was smaller than that of the left after a paired *t* test was executed (*P* = 0.006). For L5, all observed variables between let and right have no difference. These results suggest that the simulation method for standard lumbar spine radiographs is feasible and reliable in this study (Table 4).

**Table 4:** Differences between right and left for each measurement parameter.

	L3		L4		L5	
	right	left	right	left	right	left
Lateral length(mm)	16.81±2.44	17.11±2.27	17.72±3.66	17.62±3.77	18.05±4.27	18.17±3.32
AP length(mm)	4.69±0.68	4.80±0.72*	5.43±0.77	5.58±0.84*	6.56±1.03	6.64±0.93
a1(mm)	2.48±2.03	2.62±2.11	3.46±3.57	3.80±3.41	3.46±3.57	3.37±3.24
a2(mm)	4.25±2.03	4.51±1.76	6.43±3.58	6.07±3.38	5.83±3.35	5.65±3.22
a3(mm)	12.59±1.33	12.67±1.44	11.26±1.53	11.48±1.78	12.29±3.12	12.53±3.01
Estimated Lateral length(mm)	15.76±1.82	16.23±1.78*	16.21±1.84	16.42±2.13	17.01±2.86	17.03±2.68
∠α (TAN-TPA)	15.97±3.52	15.99±3.24	17.70±4.56	18.35±5.23	20.92±5.82	20.56±4.22
∠γ (TPA)	16.43±4.81	15.99±3.24	17.70±4.56	18.35±5.22	20.89±5.81	20.56±4.21
∠β (DR-TPA)	16.49±2.54	16.61±2.63	18.66±2.98	18.93±2.95	21.38±3.55	21.52±2.94

*. *P*<0.05

Moreover, the width of the pedicles (AP length) of the lower lumbar vertebrae are in the following order: L3<L4<L5. The width of the pedicle from subjects in our study is similar to that of CT data obtained from the Indian population [18]. The length of the pedicles (P2C1 = b^2+a1^2) are in the following order: L3<L4<L5. These results are consistent with the analytical feature of TPA (L3<L4<L5).

### Reliability of TPA by the TAN method

The average of the measured TPA was nearly equal to that of the TAN method, and the difference between them was not statistically significant. These results indicate that the TAN method is a very reliable way to calculate the TPA (Table 5).

**Table 5:** Reliability analysis of TPA using independent t test.

	L3-R	L3-L	L4-R	L4-L	L5-R	L5-L
∠γ (TPA)	15.97±3.51	15.99±3.24	17.70±4.56	18.35±5.22	20.92±5.82	20.56±4.22
∠α (TAN-TPA)	16.43±4.81	15.99±3.24	17.70±4.55	18.35±5.22	20.89±5.81	20.55±4.21
t	-0.622	0.002	-0.003	0.000	0.031	0.002
*P*	0.535	0.998	0.997	1.000	0.975	0.999

### Reliability of DR-TPA by measurement

An independent samples t test was used to distinguish the difference between TPA and DR-TPA, and the results showed that the difference between TPA and DR-TPA for L3, L4 and L5 was not significant (Table 6). All results show that DR-TPA could be a good method to replace the real pedicle TPA before the operation.

**Table 6:** Reliability analysis of DR-TPA using an independent t test.

	L3-R	L3-L	L4-R	L4-L	L5-R	L5-L
∠γ (TPA)	15.97±3.51	15.99±3.24	17.70±4.56	18.35±5.23	20.92±5.82	20.56±4.22
∠β (DR-TPA)	16.49±2.54	16.62±2.63	18.66±2.98	18.93±2.95	21.38±3.55	21.52±2.94
t	—0.975	-1.220	-1.443	-0.778	-0.548	-1.522
*P*	0.331	0.225	0.151	0.438	0.585	0.130

**Fig 5:**
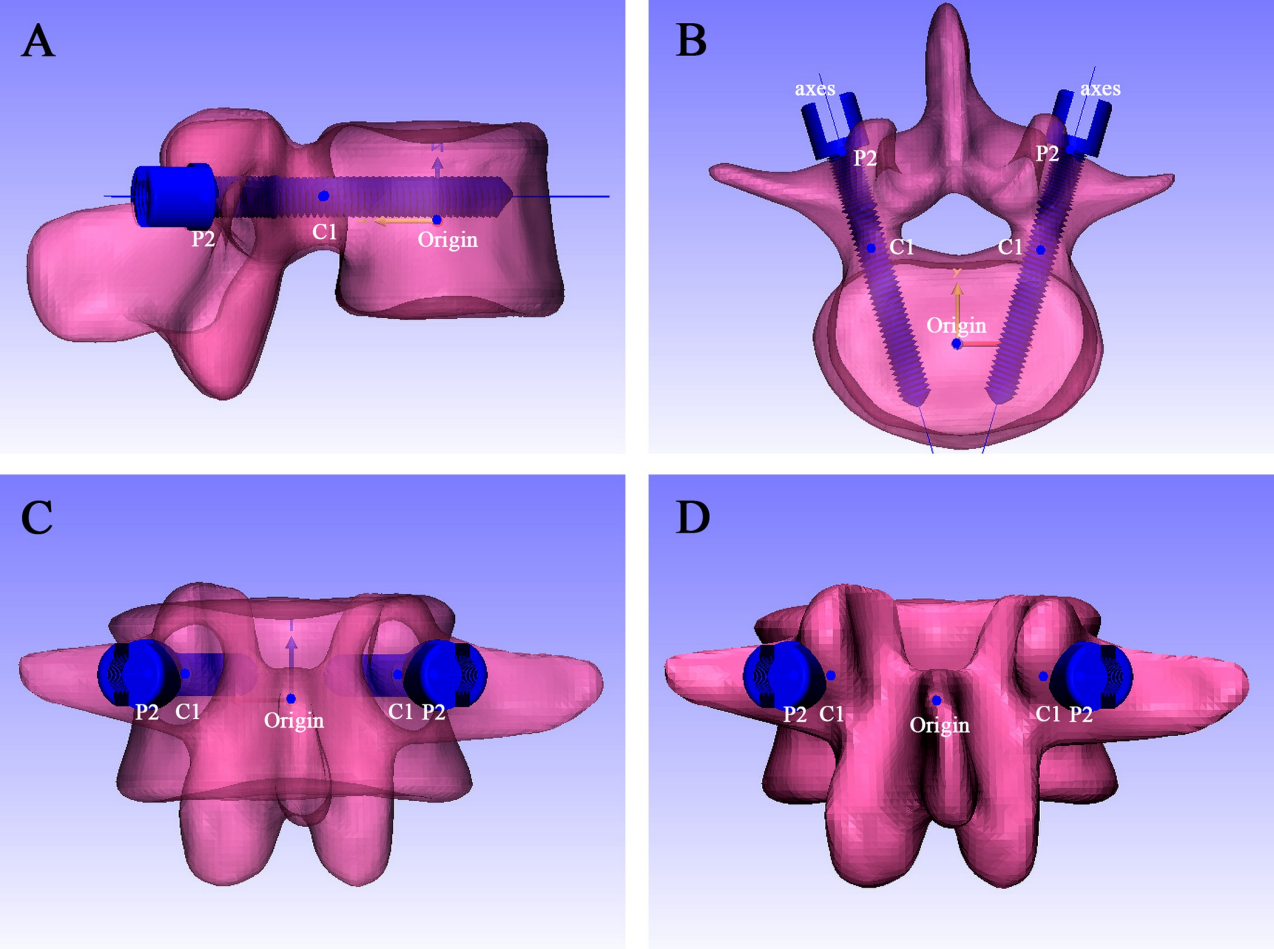
Simulated trajectory of screw placement by our approach in this manuscript (screw size:φ6.5 mm×4.5 cm). (A) view left; (B) view top; (C) view back, transparency = 0.5; (D) view back, transparency = 0.

In addition, the good location and angle of simulated screw fixation by our method are displayed in Fig 5. The real pedicle screw tunnel does not affect the anatomy of the zygapophyseal joint, and the spinal stability is not damaged by iatrogenic factors.

### Agreement analysis between TPA and DR-TPA

The intraclass correlation coefficient (ICC) was used to estimate the correlation between TPA and DR-TPA; that is, ICC was calculated to determine the reliability of the method for DR-TPA. According to published guidelines [19], the ICC of L3-R [0.815 (95% CI: 0.715–0.883)] and L3-L [0.862 (95% CI: 0.784–0.913)] indicated good reliability; moderate reliability included L4-R: 0.637 (95% CI: 0.469–0.761), L4-L: 0.622 (95% CI: 0.449–0.750), L5-R: 0.670 (95% CI: 0.512–0.784), and L5-L: 0.577 (95% CI: 0.391–0.718).

## Discussion

Spinal fixation through a pedicle screw is a classical procedure used by spine surgeons to enhance stability of the spine. DR is a routine examination that is helpful for precise intraoperative positioning. In this study, we attempted to obtain useful data from DR for determining the TPA of the lower lumbar during preoperation planning.

In recent years, some initial studies on accurate medicine have attracted attention, and many technologies have been used to improve the accuracy of pedicle screw insertion, such as computer-based navigation and robotic-assisted guidance systems [20–24], pedicle screw entry points [25–27], and TPA of the lumbar spine [17, 18, 28]. Both scan-and-plan registration and CT-to-fluoroscopy registration methods have similar fluoroscopy exposure times [29]. Nevertheless, recent reviews demonstrate that these instruments are no better than freehand technologies but also substantially increase the costs and operation time [12, 30, 31]. Overall, freehand technology under monitoring with DR is still the foremost procedure for almost all spinal surgeons. In this study, few differences were identified between true TPA and DR-TPA, and the ICC results for DR-TPA are available. AP length/estimated lateral length for L3 between right and left were statistically significant. The AP length of the right for L4 was smaller than that of the left. These positive results could be caused by developmental differences. This method is simple, practical, and feasible and has good clinical application value. Therefore, this method is worth considering for all spinal surgeries.

According to relevant reports [32], the morphology of the pedicle varies during growth. Many studies on the morphological features of the lumbar pedicle have shown some differences. The average pedicle width of normal Israel population(L3:8–9.7 mm;L4:9.8–11.5 mm;L5:14.5–16 mm) from a cross-sectional retrospective study is smaller than those in degenerative lumbar spinal stenosis (DLSS) population [33]. The transverse pedicle isthmus width from CT data of the Indian population (L3: 8.4±1.06 mm; L4: 10.1±1.18 mm; L5: 13±1.48 mm) was smaller than that in the Western population [18]. The pedicle widths of the lower lumbar vertebrae in our manuscript are similar to that of the Indian population. This could be explained by the population characteristics (body mass index/ethnicity). This can be a predictor variable for determining the diameter of the pedicle screw during the fixation operation. Additionally, the pedicle length is helpful for estimating the length of the screw.

Our calculation formula for TPA is simple, and the related parameter is easy to obtain from the DR of the lumbar vertebrae. However, DR for the lumbar spine should be standard or near the standard AP view, and it is useful for predicting or excluding breaching the medial wall of the lower lumbar pedicle [34]. According to published research [7], we also recommend using the Weinstein method for implanting pedicle screws closest to the real screw canal (Fig 5). This method in our manuscript is accepted widely by doctors generally used for preoperative planning or surgical decision-making, it maybe reduce the occurrence of complications in lumbar spine surgery.

## Conclusion

Our results demonstrate that the TPA of the lower lumbar can be calculated using the standard DR before the operation, and the estimated formula method is reliable and easy for general spinal surgeons to study. The characteristic pedicle widths of the lower lumbar vertebrae are helpful for determining the diameters of the screws, and the pedicle lengths are used for determining the lengths of the screws, which are both is critical for the safe insertion of pedicle screws.

## Supporting information

S1 Video (RAR)
